# Species Delimitation and Cryptic Diversity in *Rheotanytarsus* Thienemann & Bause, 1913 (Diptera: Chironomidae) Based on DNA Barcoding

**DOI:** 10.3390/insects16040370

**Published:** 2025-04-01

**Authors:** Yuan Yao, Jia-Yu Chen, Xiao-Ling Gong, Chen-Hong Li, Zheng Liu, Xiao-Long Lin

**Affiliations:** 1 Engineering Research Center of Environmental DNA and Ecological Water Health Assessment, Shanghai Ocean University, Shanghai 201306, China; yuanyao.work@hotmail.com (Y.Y.); chenjy.scholar@gmail.com (J.-Y.C.); xlgong@shou.edu.cn (X.-L.G.); chli@shou.edu.cn (C.-H.L.); 2 Shanghai Universities Key Laboratory of Marine Animal Taxonomy and Evolution, Shanghai Ocean University, Shanghai 201306, China; 3 Geological Museum of China, Beijing 100083, China

**Keywords:** species delimitation, *COI*, ABGD, neighbor-joining

## Abstract

The genus *Rheotanytarsus* Thienemann & Bause, 1913 (Diptera: Chironomidae) currently includes more than 120 recognized species worldwide, but precise species-level identification based solely on morphology remains challenging. Pronounced morphological differences among life stages and the time-consuming inefficiency of rearing larvae further complicate life-stage matching in this genus. In this study, we assessed species diversity by integrating morphological examination and DNA barcoding, analyzing 911 DNA barcodes from newly collected samples and a public database. Based on these results, we further constructed a relatively complete life-history framework. Our results show that 911 *Rheotanytarsus* DNA barcodes belong to 69 putative species. The maximum intraspecific divergence reached 7.35% in *R. pentapoda*, and the average minimal interspecific distance was 11.44%. Substantial intraspecific divergence in certain species complexes suggests the presence of cryptic species. Therefore, to resolve these potential cryptic species issues, more extensive sampling and morphological examination of specimens from geographically distant regions—supplemented by nuclear and ecological data—are required.

## Simple Summary

The genus *Rheotanytarsus* consists of small aquatic species valuable as indicators of undisturbed ecosystems. However, morphology-based specimen identification poses challenges, and matching life stages is difficult. This study aimed to improve the understanding of *Rheotanytarsus* species by combining traditional morphological examination with DNA barcoding, a technique that uses genetic information to distinguish species. We analyzed 911 DNA samples from both newly collected data and screened public databases, inferring that they belong to 69 different species. Our findings show that some species may be cryptic, meaning they are genetically distinct but morphologically similar. This research also provides a clearer framework for identifying these insects across different life stages, which will help in future studies of aquatic ecosystems. By enhancing species identification, our work significantly contributes to biodiversity monitoring and environmental conservation efforts. Specifically, this study improves the ability to track and protect aquatic insect populations, which are vital to ecosystem health and function. Additionally, the findings offer valuable tools for ecosystem management, particularly in assessing the impacts of environmental changes on aquatic biodiversity.

## 1. Introduction

The Chironomidae (Diptera) is one of the most abundant groups of aquatic insects in freshwater ecosystems, including over 7000 described species [1,2,3]. Chironomid larvae are widely distributed across rivers, lakes, wetlands, and other aquatic habitats worldwide [4,5,6]. Larvae exhibit a broad dietary spectrum, utilizing resources ranging from coarse particulate organic matter to dissolved organic substances, and play a pivotal role in nutrient cycling and energy transfer, significantly contributing to ecological functions [7,8,9,10,11]. Due to their widespread distribution, high species diversity, and responsiveness to environmental changes, chironomid larvae serve as key indicator organisms for freshwater assessments, playing crucial roles in pollution detection and ecosystem health evaluations [12,13,14]. However, while many chironomid genera have been extensively studied, some remain relatively underexplored, with persistent gaps in their taxonomic and ecological characterizations [15].

The genus *Rheotanytarsus* Thienemann & Bause, 1913, belonging to the tribe Tanytarsini within Chironominae, is represented by a highly diverse number of species; 120 have been described, distributed across all zoogeographic regions worldwide (except the Antarctic) [16,17,18,19,20,21,22]. Their immature stages predominantly inhabit lotic environments [22]. Notably, several species demonstrate exceptional adaptability to acidic waters, heavy metal contamination, and organic enrichment [20]. In contrast, the larvae of other species are closely associated with high dissolved oxygen levels and low phosphate concentrations, making them reliable indicators of pristine water quality [23]. *Rheotanytarsus* larvae frequently co-occur with taxa indicative of high-quality water, underscoring their potential as environmental bioindicators [20,22,24,25]. The taxonomy of this genus relies heavily on the morphological characteristics of adult male genitalia—features prone to deformation during slide preparation [19,26,27]—thus limiting the use of immature stages in environmental assessments. Moreover, pronounced morphological differences among life stages complicate the task of matching individuals [20,28], while the time-consuming and low-success rearing of larvae further limits reports on immature stages [22,29]. This hinders the full ecological understanding of the genus and limits its application in biological monitoring programs.

DNA barcoding [30,31] enhances the accuracy of identifying unknown specimens and linking life stages [32,33,34,35,36,37,38,39,40] while complementing traditional morphological methods by addressing limitations in species delimitation based on synapomorphic traits. The creation of a comprehensive barcode reference library further enriches traditional morphology, providing critical insights that deepen our understanding of biodiversity, thereby advancing integrative taxonomy [41,42]. However, issues such as heteroplasmy, nuclear mitochondrial pseudogenes (NUMTs), interspecific hybridization, and incomplete lineage sorting (ILS) limit the application of DNA barcoding in species identification [43,44]. In recent years, multilocus and phylogenomic approaches have gained increasing attention in species identification due to their ability to provide more comprehensive genetic information [45,46,47,48]. Nevertheless, DNA barcoding, as a low-cost and rapid tool, still holds significant value in large-scale preliminary identification and environmental DNA studies [49,50,51,52]. The Barcode of Life Data System (BOLD) [53], accessible at https://www.boldsystems.org/, is the world’s largest DNA barcode reference library. It serves as an essential platform for species data storage and aiding specimen identification [30,31,43,53,54,55]. In fact, DNA barcoding has contributed to taxonomic advances in several problematic taxa within Chironomidae [28,56,57,58,59,60]. Thus, for the genus *Rheotanytarsus*, for which genetic data are relatively scarce, DNA barcoding serves as an effective tool to enhance taxonomic resolution and facilitate life-history matching. However, research on DNA barcoding within this genus remains limited [61,62], and the quality of publicly available data is inconsistent. Consequently, further standardization is urgently needed to advance taxonomic progress and support its broader application in biodiversity assessments.

This study focuses on the genus *Rheotanytarsus*, integrating newly collected and publicly available DNA barcodes to address the significant molecular data gap for this genus, offering substantial practical value. This study establishes the following specific objectives: employ DNA barcoding to detect misidentifications and cryptic species potentially overlooked by traditional morphological methods; link larval, pupal, and adult stages to resolve identification challenges for immature stages and accelerate their morphological description; and contribute high-quality, standardized data to the global *COI* DNA barcode reference library for *Rheotanytarsus*, supporting subsequent molecular phylogenetic studies and taxonomic revisions. These objectives will provide a robust foundation for ecological applications and taxonomic research on *Rheotanytarsus*.

## 2. Materials and Methods

The newly added 157 specimens in this study were collected over the past decade (2008–2022) across multiple provinces in China and Namibia. During the collection process, light traps and sweep nets were used to capture adult stages, while D-nets and drift nets were employed to collect immature stages [58]. Following collection, all specimens were immediately transferred to ethanol (75% for adults, 95% for immatures) and maintained at −4 °C under dark conditions. Over recent years, we have gradually obtained DNA barcodes for all specimens. During this process, to preserve the integrity of specimens for subsequent morphological observations and to prevent DNA cross-contamination, DNA extraction and *COI* amplification were performed using the legs or thoracic muscles of adult specimens and the thoracic and abdominal muscles of larvae after gut removal. Molecular experiments and sequence processing followed established protocols [28,57,59], and the data were subsequently uploaded to BOLD. All specimens were slide-mounted in Euparal following standard protocols [63], with critical morphological structures preserved for taxonomic identification, morphometric measurements, and detailed descriptions based on established taxonomic revisions [22,64,65]. Partial results of these analyses have been published in prior studies [21,26,61,62,66]. All vouchers were deposited in the College of Fisheries and Life Science, Shanghai Ocean University, Shanghai, China.

In addition to newly acquired data, *COI* sequences ≥500 bp from BOLD were retained for *Rheotanytarsus* after excluding sequences with stop codons, BOLD-flagged quality issues, or lacking valid BINs taxonomically assigned to the genus. Data were accessed 18 November 2023. This search included datasets from two recent studies [61,62], totaling 754 sequences. Combined with the 157 newly generated sequences, this brought the total to 911 sequences. All sequences were consolidated and added to a dataset titled “Global *Rheotanytarsus COI* barcodes (DS-2023RCOI)” in BOLD.

All *COI* sequences in the dataset were downloaded in sequence page order, and alignment was performed using the BOLD Aligner. Residual primer sequences (e.g., 3′-end ′TGATTTTTT′ motif) were trimmed in Geneious Prime 2024.0.5. (https://www.geneious.com (accessed on 19 November 2024)). Following quality trimming, all retained sequences maintained ≥500 bp length in the core barcode region. Multiple sequence alignment was conducted using MUSCLE 5.1 [67] under default parameters, with the resulting alignment used to construct both Neighbor-Joining (NJ) [68] and Maximum Likelihood (ML) [69] phylogenetic trees. To ensure the robustness of species delimitation results, a combination of pairwise distance-based and phylogeny-based methods was employed. Specifically, ABGD (Automatic Barcode Gap Discovery) [70] and Neighbor-Joining (NJ) [68] analyses were used as distance-based methods, while mPTP [71] and GMYC [72] were applied as phylogeny-based techniques. For the ABGD analysis (https://bioinfo.mnhn.fr/abi/public/abgd/abgdweb.html (accessed on 19 November 2024)), the K2P model [73] was implemented with a relative gap width (X) of 1.0 and prior intraspecific divergence values ranging from 0.005 to 0.1. NJ trees were constructed in MEGA v.11 [74] using K2P distances to ensure comparability with most other DNA barcode studies on insects, with node support evaluated through 1000 bootstrap replicates. For the mPTP analysis, a maximum-likelihood (ML) tree was generated in IQ-TREE v.2.3.6 under the GTR+F+R6 model, exported in Newick format, and analyzed on the mPTP web server (https://mptp.h-its.org/#/tree (accessed on 16 March 2025)) to delimit species. For the GMYC analysis, ultrametric trees were generated in BEAST v. 2.7.6 [75] using a strict clock and HKY substitution model, with an MCMC chain length of 20 million generations. Convergence was verified using TRACER v.1. 7.2 [76], confirming that all effective sample size (ESS) values exceeded 200, validating the reliability of the results. The consensus trees were generated after discarding 10% of trees as burn-in.

Taxonomic nomenclature primarily followed morphologically validated specimens. For the 735 public sequences lacking voucher verification, outdated or invalid names were revised to reflect corresponding morphological taxa based on their clustering with validated clades. Sequences forming composite clades or exhibiting atypical intraspecific divergence were flagged and further analyzed; for groups with high or unusual intraspecific divergence, Fst values were calculated using Arlequin 3.5.2.2 [77] to assess the significance of genetic differentiation, with input files generated by DnaSP6 [78] under default settings, and these results were discussed in the context of geographic distributions and published genitalia images to evaluate potential cryptic diversity or misidentifications.

## 3. Results

### 3.1. Dataset Characteristics and Global Distribution

The 911 sequences have an average length of 617 bp, with 557 sequences (61.1%) measuring at least 600 bp, demonstrating the high completeness of the dataset. The dataset includes 109 Barcode Index Numbers (BINs), with sequences per BIN ranging from 1 to 175. Most BINs contain relatively few sequences, reflecting regional disparities in species sampling. BINs with 1–2 sequences are primarily from Southeast Asia, whereas those with more than 20 sequences are predominantly from North America and Europe, underscoring more systematic collection efforts in these regions.

The dataset encompasses three levels of taxonomic identification: Category I (Latin binomials), Category II (provisional names, e.g., *Rheotanytarsus* sp. TE01), and Category III (genus-level identification). A preliminary review of 754 publicly available *COI* sequences, combined with existing studies, identified annotation errors in 12 sequences (approximately 1.6%) under Category I. Among these, five erroneous barcodes associated with the *muscicola* species group have already been corrected in relevant revisions [61]. Although *Rheotanytarsus erignus* has been cited by submitters from multiple countries, it is not officially listed in databases such as the Catalogue of Life, ZooBank, or ITIS [16,22]. Therefore, the barcodes linked to this name were reclassified as Category III. Additionally, as *Rheotanytarsus distinctissimus* is a synonym of *R. pellucidus*, three sequences originally identified as *R. distinctissimus* were corrected [79].

From our newly collected data, morphological identification assigned 76 barcodes to Category I and 81 barcodes to Category II (labeled *Rheotanytarsus* sp. 1XL through sp. 24XL). In total, the dataset contained 364 Category I barcodes, 102 Category II barcodes, and 445 Category III barcodes. Of these, Category I barcodes covered 25 described species, exhibiting considerable variation in the number of individuals per species. Some species were represented by only a single individual, whereas as many as 196 individuals were annotated as *Rheotanytarsus ringei* (see Table S1). Category II barcodes encompassed 29 provisional names, with some annotations including the species group name (e.g., *Rheotanytarsus exiguus* group sp. 1SMRD073013-THBL038). These specimens may possess key morphological traits relevant to their respective species groups, providing valuable references for future taxonomic studies.

**Figure 1:**
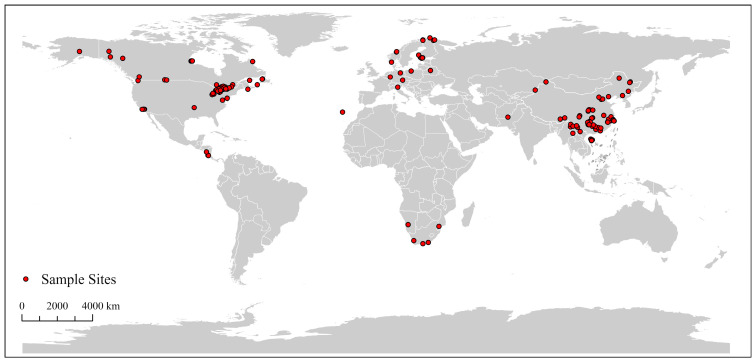
Distribution map of *Rheotanytarsus* based on the library of this study.

Barcode annotation levels vary significantly among countries, reflecting disparities in research resources, sampling strategies, and focal priorities. The dataset comprises specimens from 15 countries (Table 1 and Figure 1), covering most biogeographic regions where *Rheotanytarsus* occurs. Among Norway’s 219 sequences, 209 are Category I barcodes, accounting for an impressive 95.4%. In contrast, Canada’s 286 sequences include as many as 248 Category III barcodes, representing 86.7%. Norway, China, and the United States exhibit more active barcode research efforts compared to other countries. By contrast, barcode research activity in the Southern Hemisphere is relatively limited, with insufficient specimen collection and barcode annotation efforts.

### 3.2. Putative Species Delimitation and Genetic Divergence

**Figure 2:**
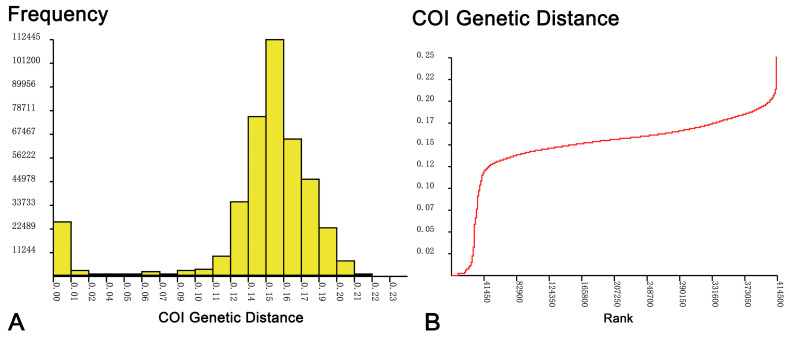
Frequency distribution of genetic distances (**A**) and ranked genetic distance curve (**B**) from the ABGD analysis of *COI* sequences for *Rheotanytarsus*.

Different species delimitation methods generated varying numbers of molecular operational taxonomic units (mOTUs). The ABGD analysis identified 71 mOTUs (Figure 2), separating *Rheotanytarsus* sp. 8XL and *R. baihualingensis* into distinct mOTUs. The NJ tree (Figure S1) clustered 911 DNA barcodes into 69 clades, with the vast majority of non-singleton clades having bootstrap values greater than 70%. Additionally, all newly collected morphospecies—verified by detailed morphological analysis—formed monophyletic groups. In the ML tree (Figure S2), most Category I and Category II barcodes formed monophyletic structures, whereas *Rheotanytarsus adjectus* and *R. miaoae* displayed paraphyletic patterns. The mPTP analysis delineated 73 mOTUs, splitting *Rheotanytarsus* sp. 8XL, *Rheotanytarsus* sp. 18XL, and *R. ashei* each into two mOTUs, while combining *R. oss*, *Rheotanytarsus* sp. 22XL, *Rheotanytarsus* sp. 23XL, and *Rheotanytarsus* sp. 24XL into one mOTU, and grouping *R. adjectus* and *R. miaoae* as a single mOTU. The GMYC analysis recognized 84 mOTUs (Figure S3), dividing *Rheotanytarsus* sp. 3XL, *Rheotanytarsus* sp. 8XL, *R. ashei*, *R. baihualingensis*, and *R. yamamotoi* each into two mOTUs. In public databases, barcodes labeled *Rheotanytarsus pentapoda* were fragmented into multiple mOTUs by both mPTP and GMYC methods, while sequences tagged as *R. curtistylus*, *R. illiesi*, *R. muscicola*, *R. pellucidus*, and *R. ringei* consistently failed to form a single mOTU across all analyses.

Apart from the previously mentioned non-monophyletic species and certain erroneous annotations discovered in the preliminary inspection, we also observed overlapping morphological identifications in two separate lineages: one containing *R. pentapoda* and *R. illiesi*, and another containing *R. muscicola* and *R. curtistylus* (the latter sharing a BIN, BOLD:ACR3994). According to the revised classification of the *muscicola* species group, some of these non-monophyletic issues can be corrected (Table S1) [61]. However, anomalously high intraspecific divergences (20.10% and 15.24%) remained in *Rheotanytarsus pellucidus* and *R. ringei*.

**Figure 3:**
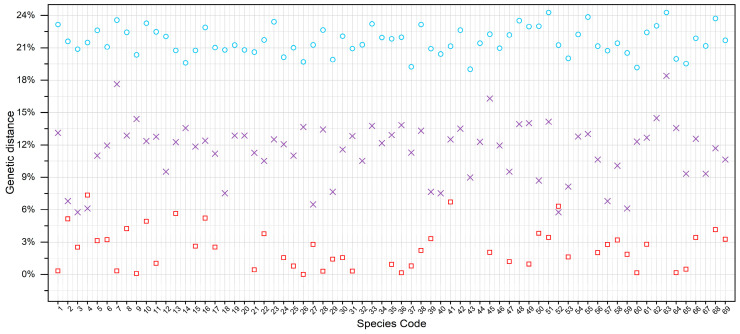
Summary of genetic distances for 69 species (K2P model): red squares (max intraspecific), purple crosses (min interspecific), blue circles (max interspecific); species codes as in Table 1.

After addressing potential cryptic species complexes, the dataset contained a total of 69 putative species (see Figure 3 and subsequent sections for details). Among these, 13 species contained only Category III barcodes, so we consequently revised the species names of the sequences in these clades from *Rheotanytarsus* sp. 1 to sp. 13 (Figure S1 and Table S1). Regarding genetic divergence, 22 species had a maximum intraspecific divergence below 2%, 18 were between 2% and 4%, 6 were between 4% and 6%, and 3 exceeded 6%. On average, the maximum intraspecific divergence was 2.43%, with the highest (7.35%) observed in *R. pentapoda*. These observations are limited by the distribution of specimens (see Discussion for details). In terms of interspecific differences, 5 species showed a minimum interspecific divergence of 6–7%, while 62 exceeded 7%. The largest interspecific genetic divergence, 24.27%, occurred between *Rheotanytarsus* sp. 4XL and *Rheotanytarsus* sp. 8, whereas the smallest, 5.77%, was detected between *Rheotanytarsus* sp. 2 and *Rheotanytarsus* sp. 9. Overall, the average minimum interspecific divergence was 11.44%.

### 3.3. Life-Stage Association and Distribution

According to the Data Spreadsheets in BOLD, we compiled life-history information for each putative species (Figure S1 and Table S2). In total, 20 species (30%) achieved successful life-stage matching, and these generally had larger sample sizes. For instance, *Rheotanytarsus ringei* (BOLD:AAF7635) was represented by 195 individuals. Among the 18 species matched for both adult and immature stages, 11 had a confirmed match between adult males and larvae. These successfully matched species provide a strong basis for subsequent integrative taxonomic studies.

By contrast, 39 species were represented solely by adults, including 32 species with adult males. Eight species consisted only of immature-stage samples: five species had larval sequences exclusively, while one species was recorded only at the pupal stage. Compared with the species that achieved complete life-stage matches, these single-stage species had relatively low sample sizes, most with no more than five specimens.

Overall, the barcoding records for *Rheotanytarsus* exhibit an uneven life-stage distribution, with most species documented primarily in the adult stage (especially males) and much scarcer data from the immature stages. This pattern likely reflects sampling biases or difficulty in collecting certain life stages. Filling these gaps through additional life-stage data and new records will help improve our understanding of *Rheotanytarsus* diversity and biology.

## 4. Discussion

### 4.1. Appropriate Delimitation Methods and Threshold Value

Single-marker species delimitation approaches demonstrate substantial methodological diversity, with accuracy being context-dependent based on dataset characteristics, necessitating both strategic selection and integrative implementation to compensate for individual method constraints [49,52,60]. Broadly, species delimitation methods based on short molecular markers can be categorized into two types: those based on genetic distance and those relying on phylogenetic criteria [54]. Our dataset displays typical limitations of single-marker studies, with singletons accounting for 30.99% (22 species) and doubletons for 15.49% (11 species)—a pattern reflecting incomplete sampling across certain lineages. These sampling gaps necessitate combining multiple delimitation methods to counteract single-method biases and minimize erroneous outcomes.

As shown in the Results, GMYC and mPTP produced mOTUs that conflicted with morphological hypotheses (particularly those confirmed by specimen examinations). This discrepancy primarily stems from the sensitivity of phylogeny-based methods to recent speciation events and undersampling, which collectively undermine phylogenetic resolution in *COI* datasets [39,80] For example, the ML tree shows paraphyly between *Rheotanytarsus adjectus* and *R. miaoae*, two morphologically distinct species described recently [26,62], requiring additional evidence to resolve their delimitation. This over-splitting tendency represents a recurrent issue in Chironomidae systematics, as evidenced by analogous discrepancies in *Tanytarsus* (2790 barcodes) [28] and *Polypedilum* (3670 barcodes) [57] studies with larger datasets, along with *Microtendipes* research (952 barcodes) [81] of comparable sampling scope. In contrast, phylogeny-based delimitation shows stronger congruence with morphology in other aquatic insects—for instance, Ephemeroptera demonstrate high GMYC-morphology concordance, suggesting clade-specific applicability of these methods [82].

**Figure 4:**
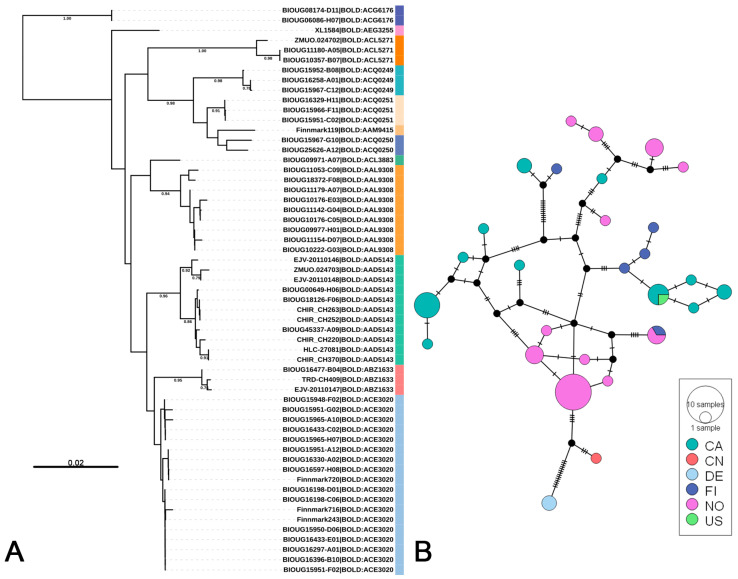
Genetic structure of *Rheotanytarsus pentapoda*. (**A**) NJ tree based on K2P genetic distances, with distinct BINs highlighted in different colors. The scale bar represents K2P genetic distances. Numbers on branches indicate bootstrap support values (>70%) based on 1000 replicates. (**B**) TCS network based on the *COI* sequences of *R. pentapoda*, where node size indicates sample size and colors represent different geographic origins (CA—Canada, CN—China, DE—Germany, FI—Finland, NO—Norway, US—United States).

Among genetic distance-based methods, the BIN system employs 2.2% as the threshold parameter for single linkage clustering [37], a value that aligns well with morphospecies boundaries in diverse animal groups [83]. However, this alignment breaks down in Chironomidae, where the same threshold significantly inflates species counts [28,56,57]. For instance, the clade of *Rheotanytarsus pentapoda*, comprising 57 individuals, spans 12 BINs in our dataset (Figure 4). ASAP, known for its operational simplicity, is widely used in species delimitation, particularly for large-scale datasets where it exhibits strong applicability; however, it is significantly affected by the number of haplotypes [80,84]. In our preliminary tests, ASAP results broadly corresponded with ABGD outcomes but demonstrated stochasticity and an over-splitting tendency. For instance, BOLD:AAH3858 and BOLD:ACJ8793 were stably merged into a single species by ABGD (*Rheotanytarsus* sp. 9), yet consistently split by ASAP. This divergence involved overseas specimens that could not be morphologically validated due to unavailable voucher data, rendering their biological interpretation inconclusive. As a foundational effort to address these data limitations, we prioritized ABGD and NJ analyses to propose preliminary species hypotheses while mitigating over-splitting risks.

No universal threshold exists owing to the variability in population sizes and divergence times among taxa [85,86], thus necessitating taxon-specific thresholds to accurately reflect biological reality. Some insects exhibit relatively low intraspecific divergence; for example, beetles and Apoidea commonly show values below 2% [87,88], Ephemeroptera display an average of around 2% [89], and a threshold of 2.5% yields optimal morphological matching in Plecoptera [90]. However, certain taxa show higher levels of intraspecific divergence, such as Psychodidae and *Smicridea* (Hydropsychidae: Trichoptera), which can reach up to 6% [91,92]. In Chironomidae, similarly high thresholds have been reported: 4–5% for *Tanytarsus* [28], 5–8% for *Polypedilum* [57], 4.5–7.7% for *Stictochironomus* [59], and a minimum interspecific threshold of 9% for *Stenochironomus* [60]. In this context, informed by our ABGD and NJ analyses alongside morphological validation, we propose a 5.77–7% intraspecific divergence threshold for *Rheotanytarsus* as suitable for species delimitation using *COI* barcodes, enabling higher-confidence estimates of putative species diversity. It is important to note that this proposed threshold is a context-specific, preliminary guideline based on our current dataset and analytical methods, and should not be interpreted as a universal standard. Furthermore, recognizing that sampling scope can influence threshold outcomes [93,94,95,96], we recommend broader and denser sampling complemented by additional species identification methods to strengthen the reliability of DNA barcode-based species delimitation.

### 4.2. Cryptic Species Diversity

Species identification is fundamental to a broad array of biological endeavors, including evolutionary research, conservation planning, and biodiversity assessments [97]. However, cryptic species present considerable challenges to assessing the species diversity of *Rheotanytarsus* and ensuring the accuracy of specimen identification [98]. In Chironomidae, abnormal *COI* divergences serve as preliminary indicators for potential cryptic diversity, triggering subsequent multilocus and morphological validation to confirm species status [99]. For instance, barcode studies in *Polypedilum* and *Tanytarsus* have supported subsequent species discoveries [56,100,101], and numerous revisions assisted by barcoding also build on *COI* differences [59,61,62], though exceptions occasionally occur [81,93]. This study offers a detailed discussion of species groups and taxa that are especially difficult to identify morphologically, integrating insights from existing taxonomic research with the anomalous intraspecific divergences observed in this study.

**Figure 5:**
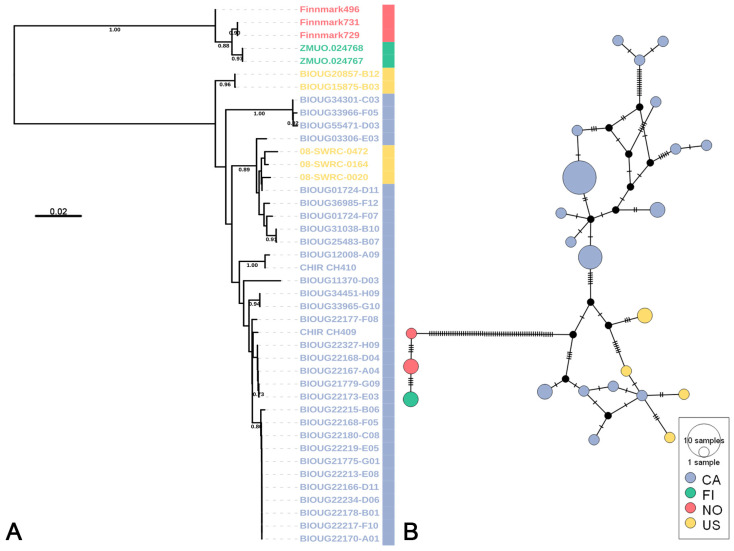
Genetic structure of *Rheotanytarsus pellucidus*. (**A**) NJ tree based on K2P genetic distances, with the scale bar representing K2P genetic distances. Numbers on branches indicate bootstrap support values (>70%) based on 1000 replicates. (**B**) TCS network based on the *COI* sequences of *R. pellucidus*, where node size indicates sample size. Colors in both panels represent different geographic origins (CA—Canada, FI—Finland, NO—Norway, US—United States).

The *Rheotanytarsus pellucidus* species group is exemplative of the taxonomic ambiguity pervasive within the genus. Morphological delineation within this group is particularly challenging due to limited descriptions of larvae and pupae across its distribution, alongside the conserved diagnostic traits of adult males, most notably the elongate Median Volsellae (MVo) with minimal interspecific variation [21,22,79]. In this study, *R. pellucidus* formed two distinct clusters in the NJ tree (Figure 5A), corresponding to populations from Europe (Finland and Norway) and North America (Canada and USA), respectively. To further elucidate their phylogeographic relationships, a haplotype network analysis was conducted (Figure 5B) and each population aligned with its corresponding cluster from the NJ tree. Significant genetic differentiation between these lineages is evidenced by a high Fst value of 0.652. Because specimens from other regions cannot be directly examined or morphologically verified, it is currently impossible to determine the precise taxonomic status of these two lineages through direct observation and measurement. Although the DNA barcode distribution of the *R. pellucidus* species complex, as documented in BOLD, corresponds to its recognized range [22], the complete absence of overlapping haplotypes and specimens from biogeographically intermediate regions underscores a stark genetic-geographic disjunction. This pattern aligns with cryptic speciation hypotheses but necessitates caution: definitive taxonomic resolution will require detailed morphological re-examinations of the MVo microstructures across isolated populations, coupled with multilocus and phylogenomic approaches.

Another example involves *Rheotanytarsus ringei*, which appears as two separate species in the NJ tree, each corresponding to a single BIN (BOLD:AAF7635 and BOLD:AAW4702). Haplotype network analysis also supports this conclusion (Figure S4). The pronounced genetic divergence between these lineages is further substantiated by an Fst value of 0.687. The marked discrepancy in the number of individuals (195 vs. 5) may reflect differences in sampling frequency or the distribution patterns of the target regions. For both lineages, some individuals have complete morphological images and detailed genitalia slide photographs uploaded to BOLD. Although the two groups share similar body coloration, they show notable differences in the anal tergal bands, gonostyli, and length of the MVo. The first group closely aligns with the original description of *R. ringei*, but further verification is needed because the characteristic notch in the MVo was not observed [102]. According to the original description, the number of hind tibial spurs in *R. ringei* exhibits intraspecific variation: the type material has two spurs, whereas Finnish specimens have only one, indicating a certain degree of variability [102]. However, most *Rheotanytarsus* species show stable numbers of posterior tibial spurs, typically two, with only a few exceptions (e.g., *R. muscicola* has one spur, but consistently) [61,62]. Furthermore, specimens (in BOLD:AAW4702) were collected from Finland and include individuals erroneously labeled as *R. muscicola*. This observation suggests that the posterior tibial spur count in that lineage is consistently one. Therefore, the second lineage (BOLD:AAW4702) may represent a cryptic species morphologically similar to *R. ringei* but characterized by a single hind tibial spur, implying that the original description could have included cryptic species. To conclusively resolve this taxonomic uncertainty, future investigations should focus on comparing immature stages, with an emphasis on pupal morphology, which may provide diagnostic traits absent in adults.

Overall, our application of DNA barcoding has advanced the taxonomic framework of *Rheotanytarsus*. While *COI* data successfully delineated most species—particularly within our newly collected samples—the paraphyly of certain morphospecies (e.g., the *R. pellucidus* and *R. ringei* complexes) underscores limitations of single-locus approaches in addressing taxonomic uncertainties. To resolve these ambiguities, especially in cases involving potential cryptic speciation, future studies must prioritize integrating morphological traits across all life stages and employing comprehensive genetic datasets for species delimitation [57,103].

### 4.3. Summary and Outlook

Our findings highlight the notable diversity of *Rheotanytarsus* in China, with 44 candidate species documented across major ecoregions. Significantly, 33 of these species constitute the first DNA barcode records from newly collected specimens, accounting for nearly half (47.8%) of all putative species identified—indicating the genus’s understudied diversity. Over half of these species remain insufficiently identified, necessitating more comprehensive studies that integrate both morphological and molecular data to clarify their taxonomic status. It is worth noting that this study encounters certain geographic sampling limitations, as the distributions of most “widespread” species appear to be primarily documented in China, Europe, and North America (Canada–USA; Table S2). These spatial constraints in specimen collection might influence our understanding of the genus’s true diversity patterns, potentially hinting at the presence of additional cryptic lineages that could be revealed through broader biogeographic sampling efforts in the future.

Species represent the fundamental units of biodiversity, and accurate specimen identification is crucial for studies in population genetics, ecology, and physiology [104,105,106,107,108]. Although reproductive isolation is a key measure for delineating species, it is often impractical to verify directly. In *Rheotanytarsus*, most species are still diagnosed by detailed examinations of adult male genital morphology, considered the “gold standard”. Yet, two major shortcomings arise in practice. First, there is no quantitative approach for measuring intraspecific variation in genitalia; if different individuals’ genital structures appear similar, even substantial variation in other morphological traits may be dismissed as mere intraspecific diversity, potentially obscuring cryptic species. Second, this method constrains the ability to link specimens across life stages, further impeding the reconstruction of complete life histories. In this study, we successfully refined the DNA barcoding database for *Rheotanytarsus*. Through the systematic application of DNA barcoding techniques, we addressed three key objectives: firstly, enhancing specimen identification and supporting species discovery; secondly, enabling life-stage associations for specimens beyond adult males; and finally, illustrating the potential of molecular methods to complement and address some of the limitations inherent in traditional taxonomic approaches.

While multilocus and phylogenomic approaches theoretically provide enhanced phylogenetic resolution, their implementation in *Rheotanytarsus* remains constrained by the insufficient genomic resources. This methodological limitation underscores the continued necessity of prioritizing *COI* barcoding as a first-step diagnostic tool in this taxon. Although the *COI* barcode demonstrates considerable utility in facilitating species discovery and delimitation within Chironomidae, its limitations as a single mitochondrial marker warrant careful consideration [109]. Firstly, hybridization between closely related species—a phenomenon confirmed within Chironomidae [110,111,112]—can lead to mitochondrial gene introgression, thereby disrupting assessments of species diversity. For instance, in the study of *Diamesa*, discrepancies between morphological identification and *COI*-based Bayesian clustering in certain individuals have been attributed to hybridization [93]. Secondly, in rapidly radiating taxa, ILS may diminish the resolving power of *COI* [113], resulting in conflicts between morphological and gene tree topologies [114]. Inter-laboratory identification discrepancies also merit attention, as they may compromise the reliability and validity of reference databases, subsequently affecting practical applications [99,115,116]. Although NUMTs have not been reported in Chironomidae [117] and genomic studies indicate their occurrence is significantly lower than in other insect groups [118], rigorous sequence quality control remains essential. In summary, scalable techniques are needed to identify and address the diversity challenges posed by “dark taxa” [119]. In the context of the rapid advancement of next-generation DNA sequencing, integrating additional genetic information—particularly nuclear genes—is crucial to resolving relationships among individuals and populations [97]. Simultaneously, standardizing morphological descriptions across all life stages, providing high-quality images, and leveraging emerging machine-assisted identification technologies can enhance specimen identification efficiency [120,121]. Equally important, for widely distributed and diverse taxa, accelerating global collaboration and the exploration of ecological traits—supported at minimum by the sharing of voucher specimen images [116]—is vital to improving the recognition of cryptic species and the resolution of rapidly radiating groups.

## 5. Conclusions

This study addresses the longstanding shortage of genetic data on *Rheotanytarsus* by employing DNA barcoding to advance its taxonomic understanding, thereby significantly enriching the existing barcode reference library and establishing a robust foundation for future specimen identification and precise aquatic ecosystem monitoring. Drawing on multiple analytical tools and morphological methods, this study allocated 911 DNA barcodes to 69 putative species and performed life-stage matching for certain taxa, thereby laying the groundwork for subsequent life-history reconstructions. Currently, *R. pentapoda* exhibits the widest distribution, whereas East Asia harbors the highest species diversity. In some species complexes, pronounced genetic divergence among individuals may indicate the presence of cryptic species, necessitating the integration of nuclear gene data, morphological traits across different life stages, and ecological information for deeper investigation. Geographic biases and life-stage gaps limit ecological inferences and may obscure true diversity patterns. While we propose that a 5.77–7% genetic threshold is more suitable for species delimitation within *Rheotanytarsus*, following the exclusion of cryptic species, its broader applicability requires rigorous testing in geographically underrepresented regions. Documenting *Rheotanytarsus* diversity demands a sustained endeavor—combining meticulous fieldwork, iterative species validation, and open-data sharing—to collectively unravel the genus’s evolutionary history and ecological roles across global scales. To address these gaps, future efforts must prioritize standardized sampling in underexplored biomes, holistic documentation of pupal and larval diagnostic traits, and consortium-driven data sharing to harmonize molecular, ecological, and morphological metadata. Collectively, these strategies will position *Rheotanytarsus* as a model for integrative taxonomy, directly enhancing the precision of freshwater biomonitoring.

## Figures and Tables

**Table 1:** Specimen Counts, Recorded Countries, and BINs for Species of the Genus *Rheotanytarsus*.

Order *	Species Identification	Specimens	Total Countries	Total BINs
1	*Rheotanytarsus* sp. 1	2	1	1
2	*Rheotanytarsus pellucidus*	37	2	6
3	*Rheotanytarsus* sp. 2	55	1	1
4	*Rheotanytarsus pentapoda*	57	6	12
5	*Rheotanytarsus* sp. 3	35	3	3
6	*Rheotanytarsus ringei*	195	3	1
7	*Rheotanytarsus* sp. 4	3	1	1
8	*Rheotanytarsus ashei*	17	1	3
9	*Rheotanytarsus* sp. 5XL	2	1	1
10	*Rheotanytarsus* sp. 5	51	1	2
11	*Rheotanytarsus* sp. 6	38	1	1
12	*Rheotanytarsus* sp. 7	1	1	1
13	*Rheotanytarsus yamamotoi*	20	1	7
14	*Rheotanytarsus diaoluoensis*	1	1	1
15	*Rheotanytarsus* sp. 11XL	3	1	1
16	*Rheotanytarsus baihualingensis*	3	1	2
17	*Rheotanytarsus muscicola*	45	5	2
18	*Rheotanytarsus curtistylus*	1	1	1
19	*Rheotanytarsus oss*	1	1	1
20	*Rheotanytarsus vallenduuki*	1	1	1
21	*Rheotanytarsus ferringtoni*	5	1	1
22	*Rheotanytarsus* sp. 19XL	28	1	1
23	*Rheotanytarsus* sp. 3XL	1	1	1
24	*Rheotanytarsus guoae*	2	1	1
25	*Rheotanytarsus* sp. 6XL	3	1	1
26	*Rheotanytarsus fluminis*	2	1	1
27	*Rheotanytarsus* sp. 2XL	11	4	1
28	*Rheotanytarsus photophilus*	5	1	1
29	*Rheotanytarsus* sp. 17XL	2	1	1
30	*Rheotanytarsus* sp. 13XL	4	1	1
31	*Rheotanytarsus yueqingensis*	3	1	1
32	*Rheotanytarsus* sp. 9XL	1	1	1
33	*Rheotanytarsus* sp. 12XL	1	1	1
34	*Rheotanytarsus* miaoae	1	1	1
35	*Rheotanytarsus* sp. 20XL	3	1	1
36	*Rheotanytarsus* sp. 14XL	9	1	1
37	*Rheotanytarsus cangshanensis*	8	1	1
38	*Rheotanytarsus* sp. 21XL	2	1	1
39	*Rheotanytarsus illiesi*	6	1	3
40	*Rheotanytarsus qiangi*	1	1	1
41	*Rheotanytarsus* sp. 24XL	3	1	3
42	*Rheotanytarsus* sp. 8XL	1	1	1
43	*Rheotanytarsus* sp. 16XL	1	1	1
44	*Rheotanytarsus* sp. 23XL	1	1	1
45	*Rheotanytarsus* sp. 15XL	3	1	1
46	*Rheotanytarsus adjectus*	1	1	1
47	*Rheotanytarsus guanacastensis*	3	2	1
48	*Rheotanytarsus* sp. 3TE	1	1	1
49	*Rheotanytarsus* sp. 2TE	5	2	1
50	*Rheotanytarsus pellucidus*	13	1	2
51	*Rheotanytarsus* sp. TE01	40	1	1
52	*Rheotanytarsus* sp. 8	38	2	2
53	*Rheotanytarsus* sp. 9	45	2	1
54	*Rheotanytarsus exiguus* group sp.	1	1	1
55	*Rheotanytarsus* sp. 7XL	1	1	1
56	*Rheotanytarsus* sp. TE-2006	31	2	1
57	*Rheotanytarsus* sp. 10	6	2	1
58	*Rheotanytarsus* sp. 11	2	1	2
59	*Rheotanytarsus* sp. 22XL	5	2	1
60	*Rheotanytarsus* cf. *ringei*	2	1	1
61	*Rheotanytarsus yui*	5	1	1
62	*Rheotanytarsus pinderi*	1	1	1
63	*Rheotanytarsus* sp. 4XL	1	1	1
64	*Rheotanytarsus* sp. 1XL	2	1	1
65	*Rheotanytarsus falcipedius*	5	1	1
66	*Rheotanytarsus* sp. 18XL	4	1	2
67	*Rheotanytarsus* sp. 10XL	1	1	1
68	*Rheotanytarsus* sp. 12	18	1	2
69	*Rheotanytarsus* sp. 13	5	1	3
	Total	911	15	109

* The species are arranged in accordance with the NJ tree (Figure S1).

## Data Availability

A list of all species, specimens, their individual images, georeferences, primers, sequences, and other relevant laboratory data of all *Rheotanytarsus* specimens are available through the public dataset “Global *Rheotanytarsus COI* barcodes (DS-2023RCOI)” in the Barcode of Life Data System (http://www.boldsystems.org).

## Supplementary Materials

The following supporting information can be downloaded at: https://www.mdpi.com/article/10.3390/insects16040370/s1, Figure S1: Neighbor-Joining tree constructed using the K2P model based on 911 *COI* barcodes. Clades of different species are marked with cyclic colors for differentiation. The scale bar represents K2P genetic distances, and numbers on branches indicate bootstrap support values (>70%) based on 1000 replicates. Solid blue circles represent specimens in immature stages, while hollow blue circles represent adult specimens. Figure S2: Maximum Likelihood (ML) tree inferred in IQ-TREE v.2.3.6 under the GTR+F+R6 model. Clades of the final putative species are marked with cyclic colors for differentiation. Numbers on branches indicate bootstrap support values (>70%) based on 1000 replicates. Figure S3: Species delimitation analysis of *Rheotanytarsus* using the GMYC single-threshold model. A. Lineage-through-time plot derived from the *COI* ultrametric tree. The red vertical line marks the transition threshold between interspecific cladogenesis and intraspecific coalescence. B. Likelihood profile of the GMYC model showing the transition peak (red dashed line) between speciation and coalescent processes. C. Ultrametric tree reconstructed in BEAST under a strict clock and HKY substitution model. Red clusters denote putative species delimited by GMYC, while black lines indicate singleton lineages. Figure S4: Genetic structure of *Rheotanytarsus ringei*. A. Neighbor-Joining tree based on K2P genetic distances, with the scale bar representing K2P genetic distances. Numbers on branches indicate bootstrap support values (>70%) based on 1000 replicates. B. TCS network based on the *COI* sequences of *R. ringei*, where node size indicates sample size. Colors in both panels represent different geographic origins (Finland—FI, Norway—NO, Poland—PL, Portugal—PT). Table S1: Dataset of 911 DNA barcodes for the genus *Rheotanytarsus*. Table S2: Individual counts based on the species delimitation results of this study, categorized by country, life stage, and BIN.
