# Supplementary figures and images for: Species Delimitation and Cryptic Diversity in Rheotanytarsus Thienemann & Bause, 1913 (Diptera: Chironomidae) Based on DNA Barcoding
Source: Insects. 2025 Apr 1;16(4):370. doi: 10.3390/insects16040370 (PMC12028281; doi:10.3390/insects16040370)

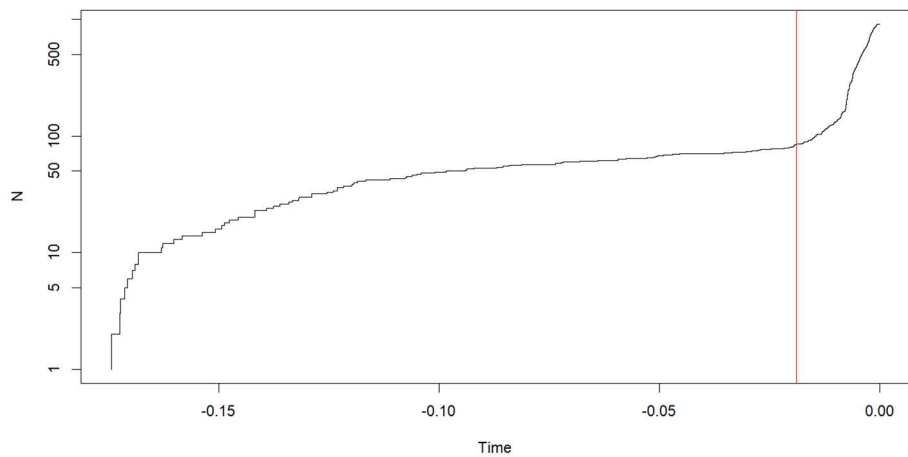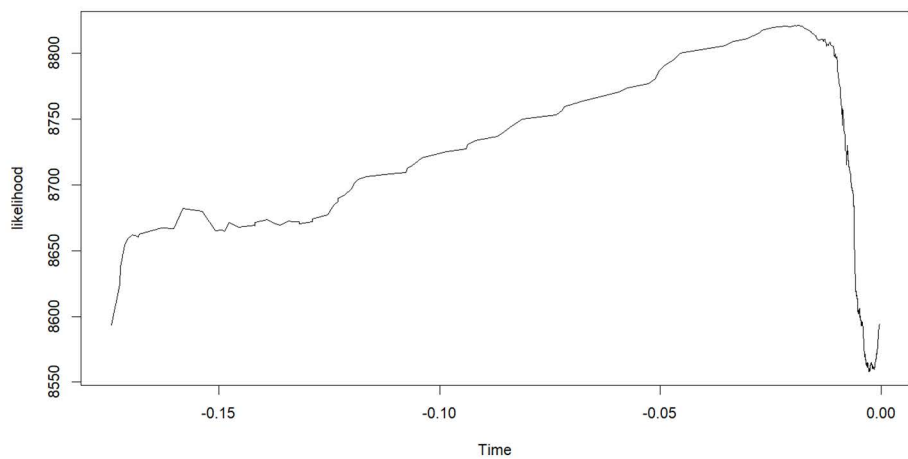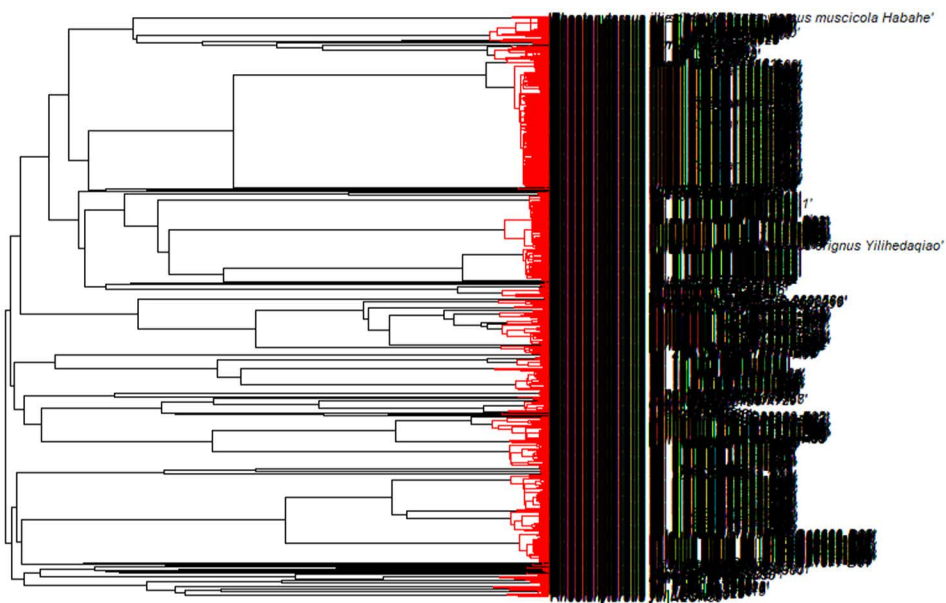

Supplement: Supplementary file 1 [file insects-16-00370-s001.zip › Figure S3.pdf]

0.02

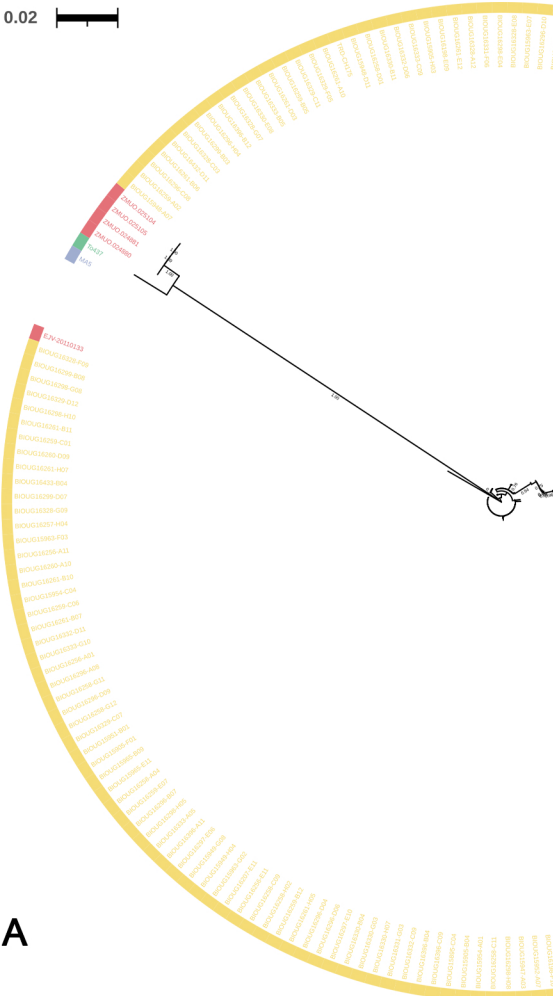

A

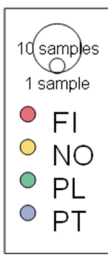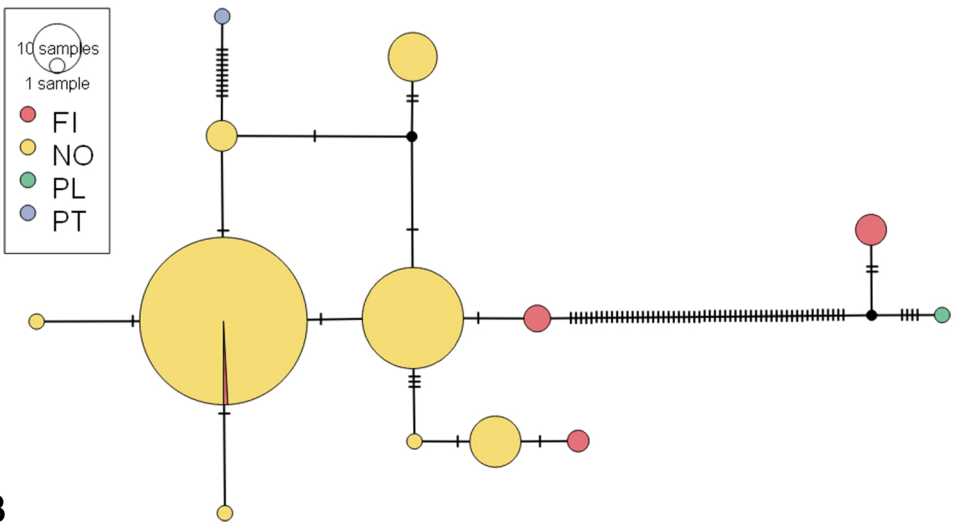

B

Supplement: Supplementary file 1 [file insects-16-00370-s001.zip › Figure S4.pdf]
