# Supplementary material for: Species Delimitation and Cryptic Diversity in Rheotanytarsus Thienemann & Bause, 1913 (Diptera: Chironomidae) Based on DNA Barcoding
Source: Insects. 2025 Apr 1;16(4):370. doi: 10.3390/insects16040370 (PMC12028281; doi:10.3390/insects16040370)
Supplement: Supplementary file 1 [file Figure S1.pdf › Figure S1.pdf]

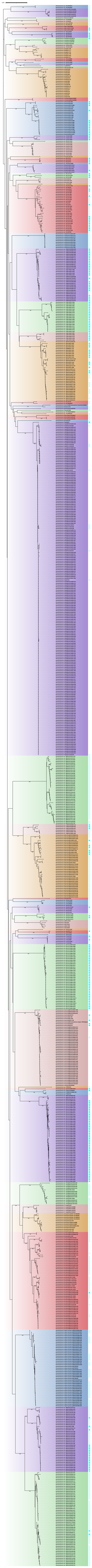
0.05
Rheotanytarsus sp. 2XL|WZSM17
Rheotanytarsus sp. 2XL|WZSM07
Rheotanytarsus pellucidus|Finnmark496
Rheotanytarsus pellucidus|ZMUO.024768
Rheotanytarsus pellucidus|ZMUO.024767
Rheotanytarsus pellucidus|Finnmark731
Rheotanytarsus pellucidus|Finnmark729
Rheotanytarsus diaoluoensis|DL03
Rheotanytarsus sp. 1XL|NAM05
Rheotanytarsus sp. 1XL|NAM3
Rheotanytarsus sp. 5XL|LSHP202
Rheotanytarsus sp. 5XL|CBL136
Rheotanytarsus adjectus|WZSM08
Rheotanytarsus adjectus|CHLA1045
Rheotanytarsus adjectus|CHLA982
Rheotanytarsus sp. 4XL|LGS257
Rheotanytarsus sp. 4|013267511
Rheotanytarsus sp. 4|013267601
Rheotanytarsus sp. 4|013267600
Rheotanytarsus yueqingensis|LDQ711
Rheotanytarsus yueqingensis|HSB30
Rheotanytarsus yueqingensis|HSY90
Rheotanytarsus yueqingensis|ZJ213
Rheotanytarsus sp. 13XL|XL3427
Rheotanytarsus sp. 13XL|XL3424
Rheotanytarsus qiangi|XL1799
Rheotanytarsus qiangi|XL1777
Rheotanytarsus qiangi|XL1791
Rheotanytarsus qiangi|XL1795
Rheotanytarsus qiangi|XL2478
Rheotanytarsus qiangi|XL2477
Rheotanytarsus sp. 22XL|HSB88
Rheotanytarsus sp. 22XL|HSB60
Rheotanytarsus sp. 23XL|XL4099
Rheotanytarsus sp. 24XL|XL1837
Rheotanytarsus oss|MHMA02
Rheotanytarsus vallenduuki|XL794
Rheotanytarsus ashei|CBL80
Rheotanytarsus ashei|ZJ405
Rheotanytarsus ashei|ZJ404
Rheotanytarsus ashei|ZJ682
Rheotanytarsus ashei|CBL135
Rheotanytarsus ashei|CBL94
Rheotanytarsus ashei|CBL144
Rheotanytarsus ashei|ZJ683
Rheotanytarsus ashei|ZJ681
Rheotanytarsus ashei|ZBT88
Rheotanytarsus ashei|CBL116
Rheotanytarsus ashei|CBL74
Rheotanytarsus ashei|ZJ077
Rheotanytarsus ashei|ZJ131
Rheotanytarsus ashei|ZJ210
Rheotanytarsus ashei|XJ59
Rheotanytarsus ashei|XJ184
Rheotanytarsus curtistylus|ZMUO.024901
Rheotanytarsus curtistylus|ZMUO.024900
Rheotanytarsus yamamotoi|ZJ460
Rheotanytarsus yamamotoi|ZJ442
Rheotanytarsus yamamotoi|CHLA980
Rheotanytarsus yamamotoi|XL1246
Rheotanytarsus yamamotoi|XL528
Rheotanytarsus yamamotoi|CHLA978
Rheotanytarsus yamamotoi|XL509
Rheotanytarsus yamamotoi|XL1243
Rheotanytarsus yamamotoi|XL1399
Rheotanytarsus yamamotoi|XL1400
Rheotanytarsus yamamotoi|XL526
Rheotanytarsus yamamotoi|CHLA938
Rheotanytarsus yamamotoi|CHLA960
Rheotanytarsus yamamotoi|CHLA981
Rheotanytarsus yamamotoi|CHLA995
Rheotanytarsus yamamotoi|CHLA996
Rheotanytarsus yamamotoi|CHLA998
Rheotanytarsus yamamotoi|CHLA1050
Rheotanytarsus yamamotoi|CHLA1046
Rheotanytarsus yamamotoi|CHLA973
Rheotanytarsus sp. 11XL|XL2280
Rheotanytarsus sp. 11XL|XL1927
Rheotanytarsus sp. 11XL|XL1928
Rheotanytarsus sp. 20XL|XL1016
Rheotanytarsus sp. 21XL|XL1304
Rheotanytarsus sp. 21XL|XL1303
Rheotanytarsus sp. 21XL|XL1793
Rheotanytarsus sp. 21XL|XL1794
Rheotanytarsus sp. 21XL|XL2474
